# First Introduction of NiSe_2_ to Anode Material for Sodium-Ion Batteries: A Hybrid of Graphene-Wrapped NiSe_2_/C Porous Nanofiber

**DOI:** 10.1038/srep23338

**Published:** 2016-03-21

**Authors:** Jung Sang Cho, Seung Yeon Lee, Yun Chan Kang

**Affiliations:** 1 Department of Materials Science and Engineering, Korea University, Anam-Dong, Seongbuk-Gu, Seoul 136-713, Republic of Korea

## Abstract

The first-ever study of nickel selenide materials as efficient anode materials for Na-ion rechargeable batteries is conducted using the electrospinning process. NiSe_2_-reduced graphene oxide (rGO)-C composite nanofibers are successfully prepared via electrospinning and a subsequent selenization process. The electrospun nanofibers giving rise to these porous-structured composite nanofibers with optimum amount of amorphous C are obtained from the polystyrene to polyacrylonitrile ratio of 1/4. These composite nanofibers also consist of uniformly distributed single-crystalline NiSe_2_ nanocrystals that have a mean size of 27 nm. In contrast, the densely structured bare NiSe_2_ nanofibers formed via selenization of the pure NiO nanofibers consist of large crystallites. The initial discharge capacities of the NiSe_2_-rGO-C composite and bare NiSe_2_ nanofibers at a current density of 200 mA g^−1^ are 717 and 755 mA h g^−1^, respectively. However, the respective 100^th^-cycle discharge capacities of the former and latter are 468 and 35 mA h g^−1^. Electrochemical impedance spectroscopy measurements reveal the structural stability of the composite nanofibers during repeated Na-ion insertion and extraction processes. The excellent Na-ion storage properties of these nanofibers are attributed to this structural stability.

Next-generation rechargeable batteries have been extensively studied as a potential power source for large-scale devices such as electric vehicles (EVs), hybrid electric vehicles (HEVs), and smart grids^1^,^2^,^3^,^4^. In addition, Na-ion batteries (NIBs) have recently received significant attention owing to their low cost and abundant global reserves compared to those of Li-ion batteries (LIBs)^5^,^6^,^7^,^8^,^9^,^10^. However, Na ion have a larger ionic radius and molar mass than Li ion; hence, NIBs exhibit lower specific capacity and rate capability, larger volume change, and shorter cycling life than LIBs^11^,^12^,^13^. Moreover, carbonaceous materials are typically used as anode materials for commercial LIBs, but their low reversible capacity and Na plating behavior render them unsuitable for NIBs^14^,^15^. The development of efficient anode materials with high reversible capacity as well as excellent cycle and high rate performance is therefore essential for the commercialization of NIBs.

Recently, metal chalcogenide materials including metal sulfides and selenides have been successfully applied as anode materials for NIBs^16^,^17^,^18^,^19^. Nanostructured metal sulfides with various compositions and their carbon composite materials have, in fact, been used in many synthesis processes, including the hydrothermal^8^,^20^,^21^, solvothermal^22^, mechanical alloying^23^, chemical vapor deposition^24^, liquid-phase exfoliation methods^25^, and spray pyrolysis^26^. However, the synthesis of nanostructured metal selenides and their carbon composite materials for use in NIBs has been scarcely studied. In one of the few studies conducted, Ko *et al*. used a spray pyrolysis process to fabricate yolk-shell-structured MoSe_2_ microspheres as an anode material for NIBs; these microspheres delivered a 50^th^-cycle discharge capacity of 433 mA h g^−1^ at a current density of 0.2 A g^−1 ^27. Zhang *et al*. used a simple hydrothermal method to fabricate nanooctahedra-assembled FeSe_2_ microspheres that delivered a stable discharge capacity of 372 mA h g^−1^ after 2000 cycles at 1 A g^−1 ^17. Furthermore, a SnSe-carbon composite formed through a facile ball milling process exhibited a high reversible capacity of 707 mA h g^−1^ at a current density of 143 mA g^−1^ (~0.2 C) and stable cycle performance over 50 cycles^19^. Carbon- and non-carbon-containing nickel sulphide materials have also been extensively studied as anode materials for NIBs^28^. However, to the best of our knowledge, nickel selenide (NiSe_2_) materials have not been investigated as anode materials for NIBs.

Electrospinning is a simple and highly versatile method used for the preparation of one-dimensional nanostructures of various compositions^29^,^30^,^31^,^32^. Therefore, various carbon- and non-carbon-containing transition metal sulfide materials have been prepared via the electrospinning process as anode materials for LIBs and NIBs^33^,^34^,^35^. Metal selenide materials designated for use in rechargeable batteries have, however, not been prepared via the electrospinning process. As such, in this study, NiSe_2_-reduced graphene oxide (rGO)-carbon composite porous nanofibers were prepared via an electrospinning and subsequent selenization process. Porous nanostructure could enhance the contact area between the host materials (NiSe_2_) and the electrolyte, additionally shorten the diffusion paths of the electrons and Na^+^ during cycling^36^,^37^. Moreover, porous structure accommodates the volume change of NiSe_2_ to alleviate pulverization during cycling^36^,^37^. Along with this, graphene has been considered as the most promising carbon matrix to support anode materials in LIBs due to its superior electrical conductivity and high mechanical strength^38^. Amorphous carbon was also incorporated into NiSe_2_-reduced graphene oxide system to effectively alleviate the aggregation of NiSe_2_ nanoparticles by separating them from each other and helped reduced graphene oxide build more-efficient 3D conducting networks. The composite nanofibers composed of Ni salt, GO nanosheets, polystyrene (PS), and polyacrylonitrile (PAN) were transformed into the NiSe_2_-rGO-C composite nanofibers by using a simple selenization process.

## Results and Discussion

**Figure 1:**
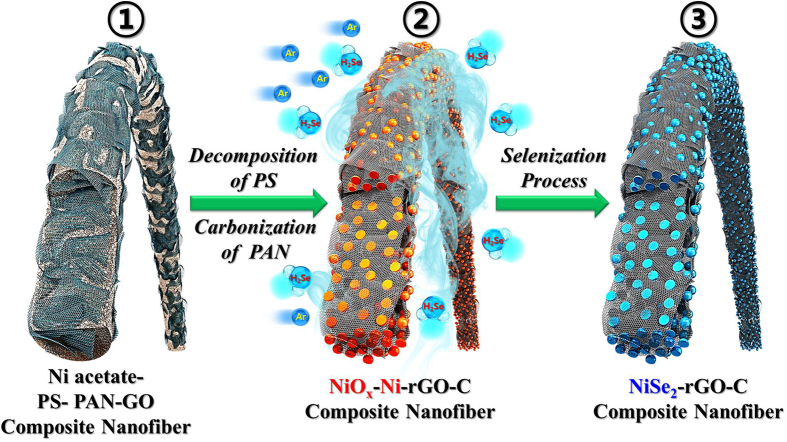
Formation mechanism of the NiSe_2_ nanoparticle-decorated rGO-C composite nanofibers.

The formation mechanism of the NiSe_2_ nanoparticle-decorated rGO-C composite nanofibers is illustrated in Fig. 1. Under the Ar atmosphere, low conductive amorphous C is produced by the carbonization of PAN and the PS is decomposed into gases. Electrospun nanofibers formed from the solution containing only PAN as an organic polymer, result therefore in C composite nanofibers with high C content. On the other hand, the PS melted before being decomposed into gases during the post-treatment of electrospun nanofibers formed from the solution containing only PS as an organic polymer; this resulted in the formation of stacked and irregular nanofibers. In this study, an optimum PS to PAN ratio of 1/4 was used to form the stable polymeric nanofibers during the post-treatment process. Highly porous NiO_x_-Ni-rGO-C composite nanofibers were formed by post-treating the electrospun nanofibers formed from the colloidal DMF solution containing Ni acetate, PS, PAN, and GO nanosheets, at 450 °C under an Ar atmosphere. Subsequent selenization of these composite nanofibers at 300 °C under H_2_Se gas gives rise to the NiSe_2_-rGO-C composite nanofibers, in which NiSe_2_ nanocrystals are uniformly distributed throughout the rGO-C composite nanofibers.

The effect of the PS on the morphologies of the electrospun nanofibers before and after the post-treatment is shown in Figs S1 and S2. As Fig. S1a shows, the electrospun nanofibers formed from the colloidal DMF solution containing Ni acetate, PS, PAN, and GO nanosheets have a uniform morphology. The electrospun nanofibers formed from the DMF solution containing Ni acetate and PAN only also had a uniform morphology (Fig. S2a). Furthermore, owing to the elimination of PS via decomposition, nanofibers having a porous wood pulp, fiber-like structure formed when the electrospun nanofibers containing GO nanosheets were post-treated under an Ar atmosphere; the formation of the porous structure was also affected by the nanosheets. However, the electrospun nanofibers formed from the DMF solution containing Ni acetate and PAN only, had a uniform morphology even after a post-treatment at 450 °C under air atmosphere. The corresponding XRD pattern (Fig. S2c) revealed that phase-pure NiO nanofibers formed after this post-treatment.

**Figure 2:**
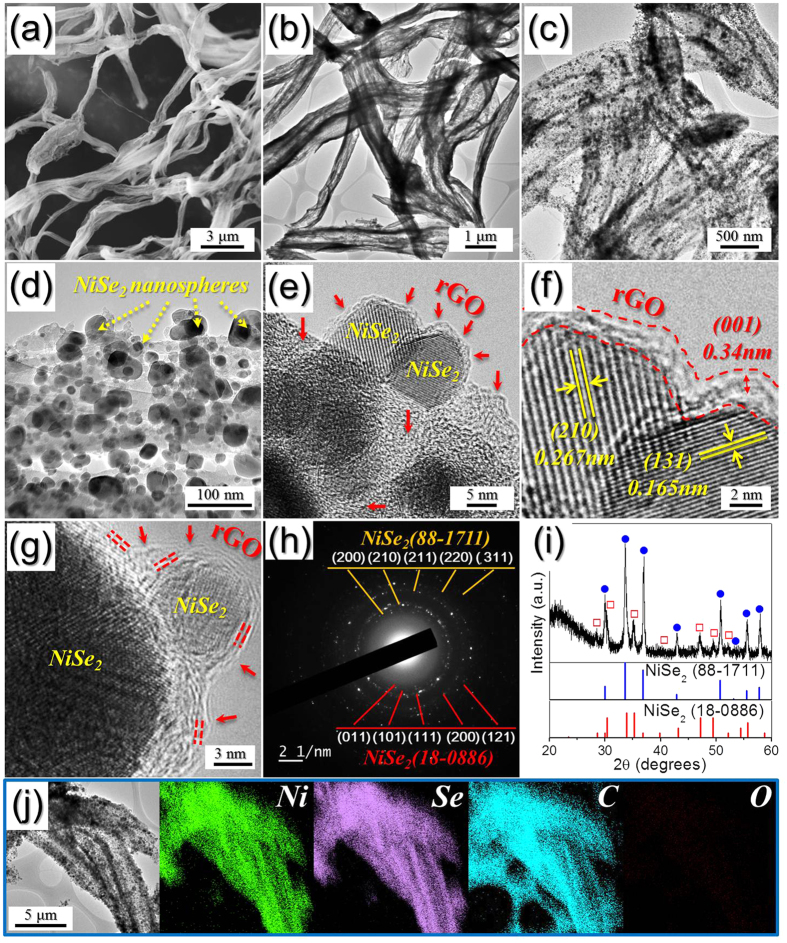
Morphologies, SAED pattern, XRD pattern, and elemental mapping images of the NiSe_2_-rGO-C composite nanofibers formed by selenization process of the carbonized nanofibers. (**a**) SEM image, (**b**–**d**) TEM images, (**e**–**g**) HR-TEM images, (**h**) SAED pattern, (**i**) XRD pattern, and (**j**) elemental mapping images.

Figure 2 shows the morphologies of the NiSe_2_-rGO-C composite nanofibers resulting from the selenization of the nanofibers shown in Fig. S1. As Fig. 2a,b show, the wood pulp fiber-like porous structure of the nanofibers persists even after the selenization process. The low-resolution TEM images (Fig. 2c,d) revealed that ultrafine NiSe_2_ nanocrystals are uniformly distributed over the surface of the composite nanofibers. These nanocrystals have a single-crystalline structure (Fig. 2e,f), as revealed by the high-resolution images. They also exhibit clear 0.267- and 0.165-nm-spaced lattice fringes, which correspond to the (210) and (131) crystal planes of the cubic and orthorhombic NiSe_2_ phases, respectively; stacked multi-layer rGO was also observed in the images, as indicated by the arrows in Fig. 2e,g. In addition, the SAED and XRD patterns (Fig. 2h,i) indicate that the NiSe_2_-rGO-C composite nanofibers have mixed crystal structures of cubic and orthorhombic NiSe_2_ phases. The broad XRD peaks shown in Fig. 2i are attributed to the formation of the ultrafine NiSe_2_ nanocrystals that have a mean size of 27 nm, as determined from the TEM images. Furthermore, energy dispersive spectroscopy (EDS) analysis revealed that the composite nanofibers (Fig. S3) had a Ni-to-Se component ratio of ∼2. The corresponding elemental mapping images (Fig. 2j) show that the NiSe_2_ nanocrystals are uniformly distributed throughout the rGO-C matrix, as revealed previously by the TEM images. Moreover, D and G bands corresponding to graphene occur at 1340 and 1605 cm^−1^, respectively, in the Raman spectrum (Fig. S4). The higher signal peak intensity of the D band compared to that of the G band, is indicative of the thermal reduction of GO nanosheets to rGO nanosheets during the two-step post-treatment process. In addition, carbonization of the PAN and consequent formation of amorphous C resulted in an increase in the intensity of the peak corresponding to the D band. The TG curve of the NiSe_2_-rGO-C composite nanofibers shown in Fig. S5 revealed a three-step weight loss for temperatures below 600 °C. The weight loss observed at temperatures between 350 and 600 °C was attributed to the decomposition of NiSe_2_ into NiO and the combustion of rGO and amorphous C. The rGO and amorphous C contents of 25% was estimated from the TG analysis of the NiSe_2_-rGO-C composite nanofibers. The chemical state and molecular environment of the NiSe_2_-rGO-C composite nanofibers were characterized via X-ray photoelectron spectroscopy (XPS). The resulting XPS survey spectrum (Fig. S6) exhibited Ni, Se, and C signals. In fact, the main peaks of the Ni 2p spectrum (Fig. S6a) occurred at binding energies of 853.5 eV and 873.4 eV corresponding to Ni 2p_3/2_ and Ni 2p_1/2_, respectively; these are characteristic of NiSe_2_ and two shake-up satellites. A peak at a binding energy of 54.5 eV in the Se 3d spectrum (Fig. S6b) also confirmed the presence of NiSe_2_ in the composite nanofibers, in accordance with previous studies^39^. Furthermore, the C 1s spectrum (Fig. S6c) exhibits peaks corresponding to sp^2^-bonded carbon (C–C), epoxy and alkoxy groups (C–O), and carbonyl and carboxylic (C=O) components at 284.6, 286.6, and 288.1 eV, respectively; the peak corresponding to the C–C bond exhibits high intensity, whereas those associated with the C–O and C=O bonds have low intensities. This indicates that the GO nanosheets undergo thermal reduction to rGO nanosheets during the two-step post-treatment preparation process^40^,^41^.

**Figure 3:**
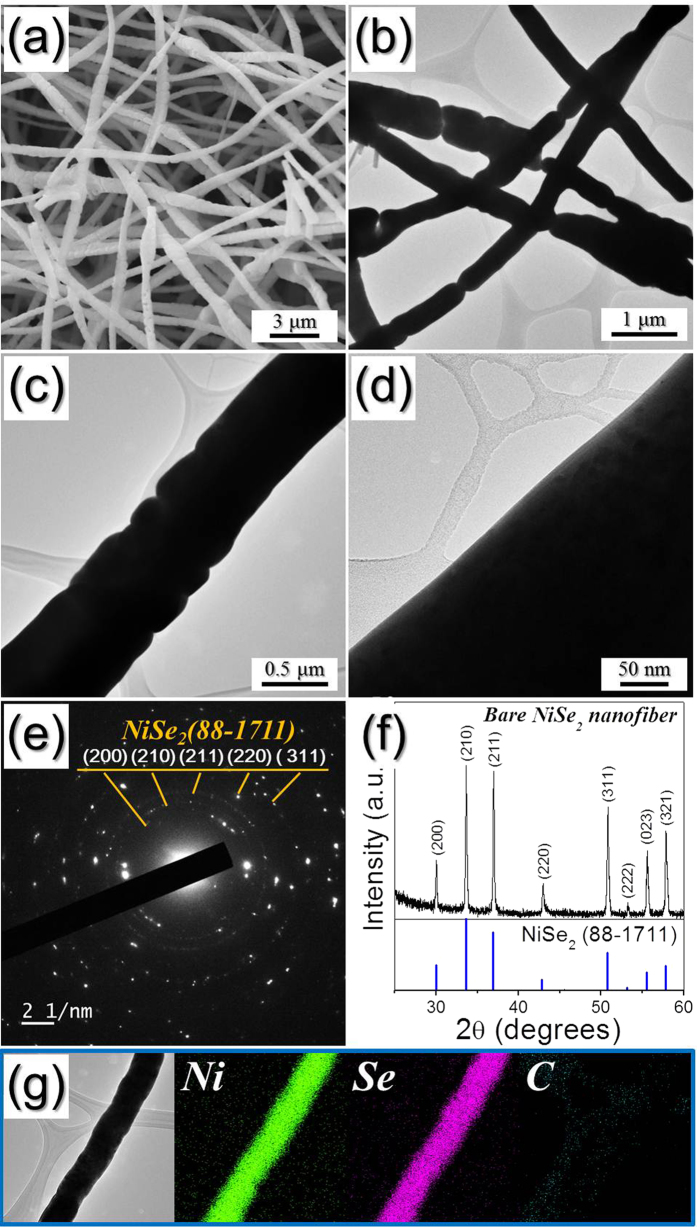
Morphologies, SAED pattern, XRD pattern, and elemental mapping images of the bare NiSe_2_ nanofibers formed by selenization process of the nanofibers. (**a**) SEM image, (**b**–**d**) TEM images, (**e**) SAED pattern, (**f**) XRD pattern, and (**g**) elemental mapping images.

The SEM and TEM images (Fig. 3) show the morphologies of the bare NiSe_2_ nanofibers formed via selenization of the nanofibers shown in Fig. S2; these densely structured (i.e., non-porous) nanofibers are formed when pure NiO nanofibers are selenized. Moreover, the SAED and XRD patterns (Fig. 3e,f) reveal that the NiSe_2_ nanofibers are cubic and phase-pure (i.e., do not contain impurity phases). The elemental mapping images also (Fig. 3g) confirm that these NiSe_2_ nanofibers, formed via the two-step post-treatment process of the Ni acetate-PAN electrospun nanofibers, are C-free. The BET surface areas of the bare NiSe_2_ and NiSe_2_-rGO-C composite nanofibers were 21 and 119 m^2^ g^−1^, respectively (Fig. S7).

**Figure 4:**
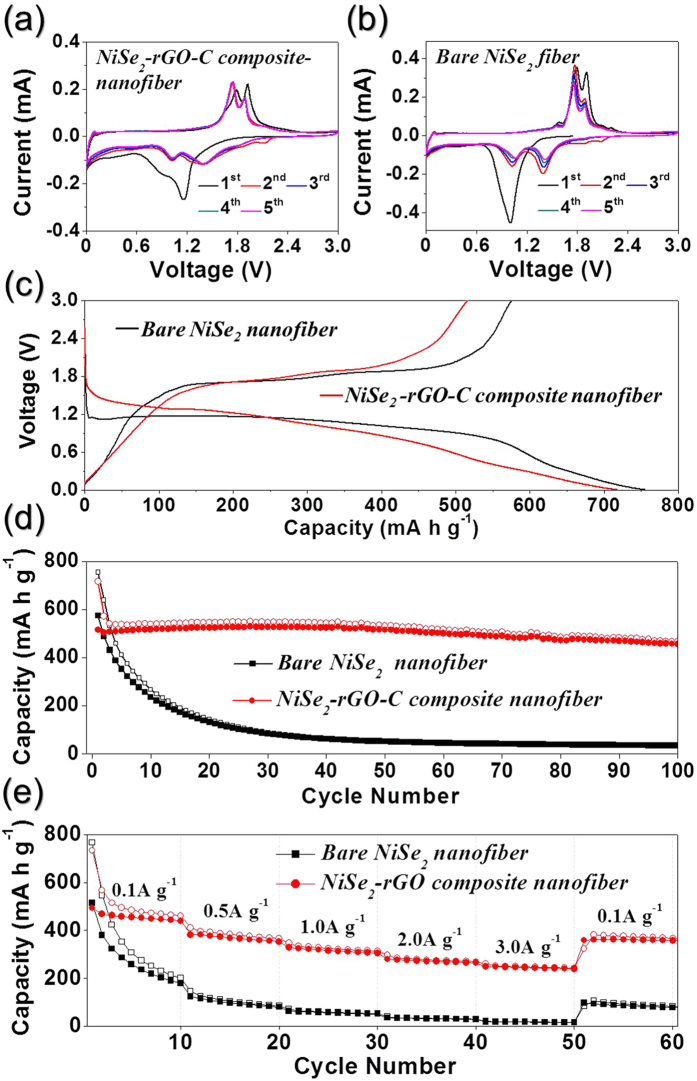
Electrochemical properties of the NiSe_2_-rGO-C composite nanofibers and dense structured bare NiSe_2_ nanofibers for sodium ion storage. (**a**) cyclic voltammogram (CV) curves of NiSe_2_-rGO-C composite nanofibers, (**b**) CV curves of bare NiSe_2_ nanofibers, (**c**) initial charge/discharge curves at a constant current density of 200 mA g^−1^, (**d**) cycling performances at a constant current density of 200 mA g^−1^, and (**e**) rate performances.

The electrochemical properties of the NiSe_2_-rGO-C composite nanofibers for Na-ion storage were compared with those of the densely structured NiSe_2_ nanofibers; this comparison was made via cyclic voltammograms (CVs) and galvanostatic discharge/charge cycling at voltages ranging from 0.001–3 V vs. Na/Na^+^. Figure 4a,b show the CV curves obtained at a scan rate of 0.07 mV s^−1^ from the first five cycles of the NiSe_2_-rGO-C composite and bare NiSe_2_ nanofibers, respectively. The first cathodic scan of the NiSe_2_-rGO-C composite nanofibers exhibited two broad peaks at 1.2 and 0.9 V. However, the Na-ion insertion and desertion mechanisms of NiSe_2_ have not been identified in previous studies and therefore, the electrochemical reaction of NiSe_2_ for Na-ion storage was estimated from that of NiS^28^. The reaction of NiS with Na proceeds as follows: NiS + 2Na ↔ Na_2_S + Ni^28^. The reduction peaks located at 1.2 and 0.9 V may therefore be attributed to the formation of NiSe_x_ and Na_2_Se, and Ni and Na_2_Se, respectively. During the anodic scans, two oxidation peaks, which may be attributed to the formation of NiSe_x_ and NiSe_2_, occurred at respective voltages of 1.8 and 1.9 V. A broad reduction peak (Fig. 4b) occurs at a potential of ∼1.0 V during the first cathodic scan of the bare NiSe_2_ nanofibers. However, the reduction peaks of both the bare and composite nanofibers shifted to higher voltages than those associated with the first cycle; these shifts resulted from the improved kinetics of the electrode owing to the formation of ultrafine nanoclusters after the first cycle. The significant overlapping of the CV curves from the second cycle onward (Fig. 4a) indicated the excellent cycling stability of the NiSe_2_-rGO-C composite nanofibers. In contrast, the intensities of the peaks in the reduction and oxidation curves (Fig. 4b) of the bare NiSe_2_ nanofibers decreased during the first five cycles. The initial discharge and charge curves obtained at a current density of 200 mA g^−1^ are compared in Fig. 4c. The discharge curve of the bare NiSe_2_ nanofibers exhibits a clear plateau at ∼1.2 V, whereas that of the NiSe_2_-rGO-C composite nanofibers decreases monotonically without clear plateaus. The different crystallite sizes of the two samples changed the shapes of the initial discharge curves. The bare and composite nanofibers consist of coarse and fine crystallites, respectively. They also exhibit respective initial discharge capacities of 717 and 755 mA h g^−1^, and charge capacities of 516 and 575 mA h g^−1^. The rGO and amorphous C with low Na-ion storage capability led to a reduction in the initial discharge capacity and Coulombic efficiency of the composite nanofibers. Fig. 4d shows the cycling performance of the nanofibers at a current density of 200 mA g^−1^; the discharge capacities of the NiSe_2_-rGO-C nanofibers remained at high levels for 100 cycles, whereas those of their bare NiSe_2_ counterparts decreased sharply to 90 mA h g^−1^ during the first 30 cycles. In fact, the former and latter had 100^th^-cycle discharge capacities of 468 and only 35 mA h g^−1^, respectively. The rate performances of the both samples are shown in Fig. 4e, in which the current density is increased step-wise from 0.1 to 3.0 A g^−1^. The NiSe_2_-rGO-C composite nanofibers had superior rate capability compared to that of the bare NiSe_2_ nanofibers. As the figure shows, the composite nanofibers had final discharge capacities of 461, 365, 313, 269, and 243 mA h g^−1^ at current densities of 0.1, 0.5, 1.0, 2.0, and 3.0 A g^−1^, respectively. These capacities were almost recovered to 383 mA h g^−1^ when the current density was returned to 0.1 A g^−1^ after cycling at high current densities.

**Figure 5:**
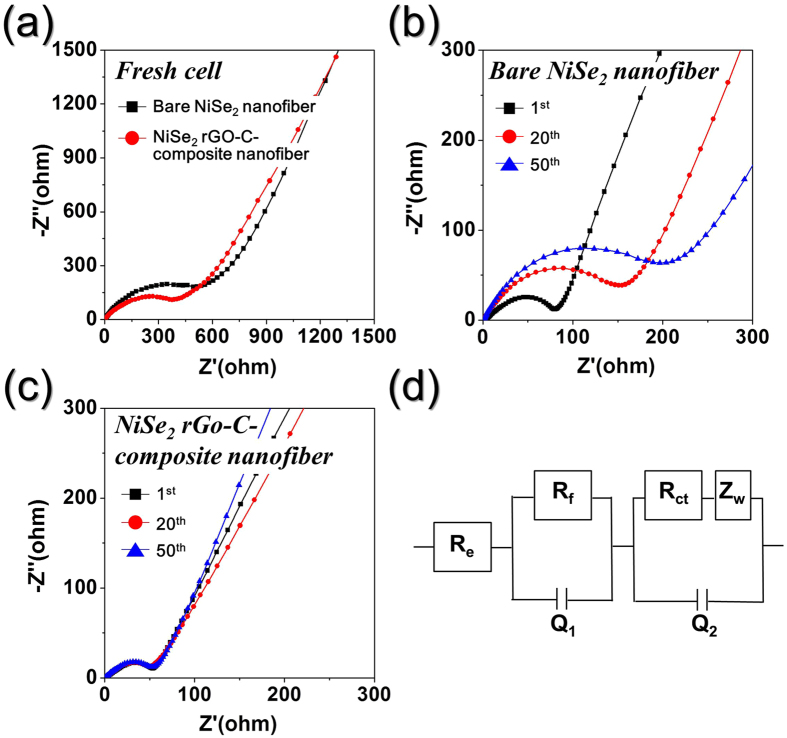
Nyquist impedance plots of the NiSe_2_-rGO-C composite nanofibers and bare NiSe_2_ nanofibers. (**a**) Nyquist impedance plots of the nanofibers before cycling, (**b**) Nyquist impedance plots of bare NiSe_2_ nanofibers after cycling, (**c**) Nyquist impedance plots of NiSe_2_-rGO-C composite nanofibers after cycling, and (**d**) equivalent circuit model used for AC impedance fitting.

The basis for the superior Na-ion storage properties of the composite nanofibers compared to those of their bare counterparts was determined by performing EIS measurements before and after cycling of the nanofibers. These measurements were performed at room temperature before and after 1, 20, and 50 cycles at a current density of 200 mA g^−1^. The resulting Nyquist plots (Fig. 5) are characterized by semi-circles in the medium-frequency range, which are attributed to the charge-transfer resistance (*R*_*ct*_) of the electrodes^42^,^43^. The precise values of *R*_*ct*_ are calculated from a simulated equivalent circuit. Prior to cycling, the porous highly conductive NiSe_2_-rGO-C composite nanofibers had a lower *R*_*ct*_ of 373 Ω (Fig. 5a) than 587 Ω of the bare NiSe_2_ nanofibers. The synergetic effect of the porous nanostructure and a graphitic carbon resulted in the low *R*_*ct*_ of the NiSe_2_-rGO-C composite nanofibers. The *R*_*ct*_ of NiSe_2_-rGO-C composite and bare NiSe_2_ nanofibers decreased to 53 and 81 Ω after the first cycle, respectively, owing to transformation of the NiSe_2_ crystals into ultrafine nanocrystals during first cycling^44^. In contrast, the *R*_*ct*_ of the bare NiSe_2_ nanofibers increased significantly from 155 to 210 Ω as the number of cycles increases from 20^th^ to 50^th^ cycle, as shown in Fig. 5b. However, the *R*_*ct*_ of the NiSe_2_-rGO-C composite nanofibers remained approximately constant (Fig. 5c) for 50 cycles. These results are indicative of the structural stability of the NiSe_2_-rGO-C composite nanofibers during repeated Na^+^ insertion and extraction processes. In contrast, the loss of structural stability during cycling increased the *R*_*ct*_ values of the bare NiSe_2_ nanofibers.

**Figure 6:**
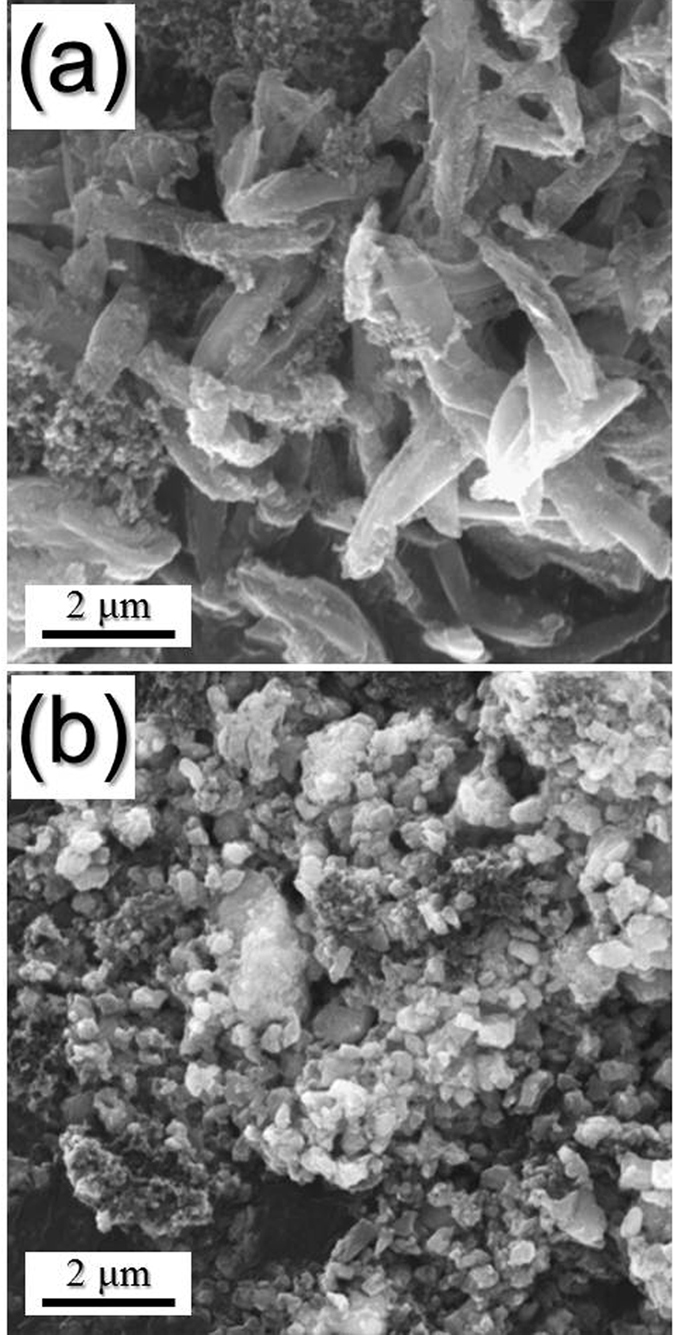
Morphologies of the (**a**) NiSe_2_-rGO-C composite nanofibers and (**b**) bare NiSe_2_ nanofibers with dense structure obtained after 100 cycles at a constant current density of 200 mA g^−1^.

To support this proof obviously, the morphologies of the NiSe_2_-rGO-C composite nanofibers and densely structured NiSe_2_ nanofibers obtained after 100 cycles are shown in Fig. 6. The NiSe_2_-rGO-C composite nanofibers maintained their original morphologies even after repeated sodium ion insertion and desertion processes as shown by Fig. 6a. In contrast, the structure of the bare NiSe_2_ nanofibers with dense structure was broken into several pieces after cycling in Fig. 6b. The destruction of the solid NiSe_2_ nanofibers during cycling decreased the electrical contact with the copper foil electrode. The excellent Na-ion storage properties of the NiSe_2_-rGO-C composite nanofibers are therefore attributed to this structural stability.

## Conclusions

The first-ever investigation of NiSe_2_ materials for use in Na-ion batteries was conducted in this work. Porous-structured NiSe_2_-rGO-C composite nanofibers with appropriate amounts of amorphous C were prepared by optimizing the composition of the organic polymers used in the electrospinning process. The high density of polystyrene, which could be decomposed into gases even under an Ar atmosphere, enabled the formation of porous-structured NiSe_2_-rGO-C composite nanofibers. These nanofibers were prepared via electrospinning and a subsequent selenization process and exhibited superior Na-ion storage properties compared to those of the bare densely structured NiSe_2_ nanofibers. The synergetic effect of the porous structure of the composite nanofibers and the highly electrically conductive rGO-C matrix improved the electrochemical properties of the NiSe_2_-rGO-C composite nanofibers for Na-ion storage applications. More importantly, the process developed in this study is applicable to both carbonaceous and non-carbonaceous metal selenide materials that can be used in various applications, including rechargeable secondary batteries.

## Materials and Methods

### Sample preparation

NiSe_2_-reduced graphene oxide (rGO)-C composite nanofibers were prepared in a three-step process. During this process, nickel (II) acetate tetrahydrate-polyacrylonitrile-polystyrene with graphene oxide (GO) [Ni(OCOCH_3_)_2_·4H_2_O-PAN-PS-GO] composite nanofibers were prepared as precursor fibers via electrospinning. GO was synthesized from graphite flakes using a modified Hummers method, as described in our previous report^36^. The precursor solution for the electrospinning process was prepared through vigorous overnight stirring of 1.5 g of Ni(OCOCH_3_)_2_·4H_2_O (Aldrich, 99%), 1 g of PAN (polyacrylonitrile, Aldrich, M_w_: 150,000), 4 g of PS (polystyrene, Aldrich, M_w_: ~192,000), and 0.2 g of GO into a solution of 50 mL of N,N-dimethylformamide (DMF, Aldrich, 99%). The prepared solution was loaded at a flow rate of 2 mL h^−1^ into a plastic syringe equipped with a 25-gauge stainless steel nozzle, ejected, and subsequently electrospun onto a drum collector covered with Al foil. During the electrospinning process, the drum rotated at 100 rpm and a distance of 20 cm was maintained between the tip and the collector; a voltage of 25 kV was applied between the collector and the syringe tip. The first step in the post-treatment process was performed at 450 °C for 3 h under an Ar atmosphere, and yielded NiO_x_-Ni nanosphere-decorated rGO-C composite nanofibers. In the second step of the process, these composite nanofibers were subjected to selenization at 300 °C for 10 h in H_2_Se gas, which was formed from commercial Se metal powders and H_2_ gas. For the selenization process, the composite nanofibers and Se metal powders were loaded into a covered alumina boat and placed in a quartz tube reactor; selenization resulted in the formation of NiSe_2_ nanosphere-decorated rGO-C composite nanofibers. Bare NiSe_2_ nanofibers were also synthesized for the sake of comparison. The nanofibers were prepared by electrospinning Ni(OCOCH_3_)_2_·4H_2_O-PAN composite nanofibers, without PS and GO, under conditions identical to the aforementioned conditions. Bare NiSe_2_ nanofibers were obtained by post-treating the resulting Ni(OCOCH_3_)_2_·4H_2_O-PAN composite nanofibers at 450 °C for 3 h under an air atmosphere and then selenizing at 300 °C for 10 h in H_2_Se gas.

### Characterization

The microstructure of the nanofibers was examined via field emission scanning electron microscopy (SEM; Hitachi, S-4800) and field emission transmission electron microscopy (TEM; JEOL, JEM-2100F). In addition, X-ray diffraction (XRD; X’Pert PRO MPD) using Cu K_α_ radiation (λ = 1.5418 Å), performed at the Korea Basic Science Institute (Daegu), was used to evaluate the corresponding crystal structures. The composition of the specimens was determined via X-ray photoelectron spectroscopy (XPS; Thermo Scientific K-Alpha) performed with a focused monochromatic Al Kα beam operating at 12 kV and 20 mA. Moreover, the surface area of the nanofibers was determined by using the Brunauer–Emmett–Teller (BET) method, with N_2_ as the adsorbate gas. The structure of the C in the specimens was characterized via Raman spectroscopy (Jobin Yvon LabRam HR800, excitation source: 632.8-nm He-Ne laser) performed at room temperature. Furthermore, thermogravimetric analysis (TGA) was performed using a Pyris 1 TGA (Perkin Elmer) at temperatures ranging from 25–650 °C at a heating rate of 10 °C min^−1^ under a static air atmosphere.

### Electrochemical measurements

The electrochemical properties of the fabricated NiSe_2_ nanofibers were determined by constructing a 2032-type coin cell. The anode was prepared by mixing the active material, carbon black, and sodium carboxymethyl cellulose (CMC) in a weight ratio of 7:2:1. Na metal and microporous polypropylene film were used as the counter electrode and the separator, respectively. Moreover, the electrolyte used consisted of 1 M NaClO_4_ (Aldrich) dissolved in a mixture of ethylene carbonate/dimethyl carbonate (EC/DMC; 1:1 v/v), to which 5 wt% fluoroethylene carbonate (FEC) was added. The discharge/charge characteristics of the samples were investigated by cycling the cells at various current densities for potentials ranging from 0.001–3 V. The corresponding cyclic voltammograms (CV) were measured at a scan rate of 0.07 mV s^−1^. The dimensions of the negative electrode were 1.5 cm × 1.5 cm and the mass loading was approximately 1.2 mg cm^−2^. In addition, the electrochemical impedance over a frequency range of 0.01 Hz–100 kHz was measured via electrochemical impedance spectroscopy (EIS).

## Additional Information

**How to cite this article**: Cho, J. S. *et al*. First Introduction of NiSe_2_ to Anode Material for Sodium-Ion Batteries: A Hybrid of Graphene-Wrapped NiSe_2_/C Porous Nanofiber. *Sci. Rep*. **6**, 23338; doi: 10.1038/srep23338 (2016).

## Supplementary Material

Supplementary Information
