# Supplementary material for: First Introduction of NiSe2 to Anode Material for Sodium-Ion Batteries: A Hybrid of Graphene-Wrapped NiSe2/C Porous Nanofiber
Source: Sci Rep. 2016 Mar 21;6:23338. doi: 10.1038/srep23338 (PMC4800391; doi:10.1038/srep23338)
Supplement: Supplementary Information [file srep23338-s1.pdf]

Supporting Information

First Introduction of $NiSe_2$ to Anode Material for Sodium-Ion Batteries: A Hybrid of Graphene-Wrapped $NiSe_2$/C Porous Nanofiber

*Jung Sang Cho, Seung Yeon Lee, and Yun Chan Kang**

J. S. Cho, Y. L. Lee, and Prof. Y. C. Kang

Department of Materials Science and Engineering

Korea University

Anam-Dong, Seongbuk-Gu

Seoul 136-713, Republic of Korea

E-mail: *yckang@korea.ac.kr*

Keywords: Nickel selenide, sodium ion batteries, graphene, carbon composite, electrospinning

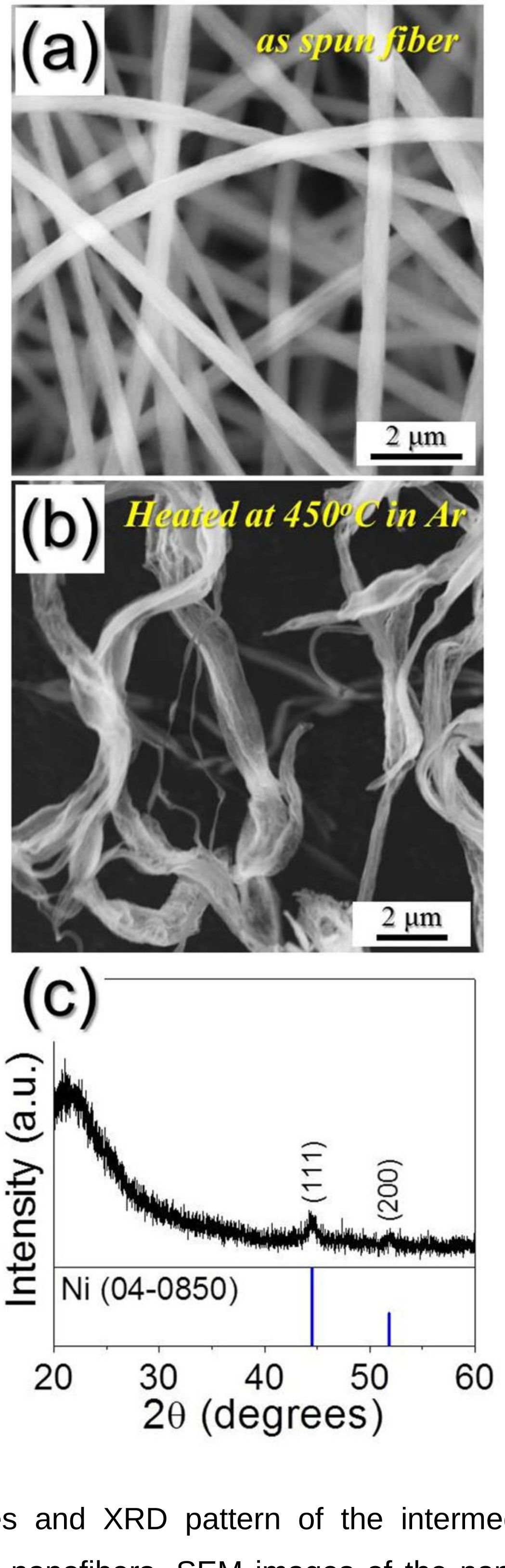

Figure S1 Morphologies and XRD pattern of the intermediate structures of the $NiSe_2$-rGO-C composite nanofibers. SEM images of the nanofibers (a) before heat treatment, (b) after heat treatment at 450 ^{o}C in Ar, and (c) XRD pattern of the nanofibers after heat treatment at 450 ^{o}C in Ar.

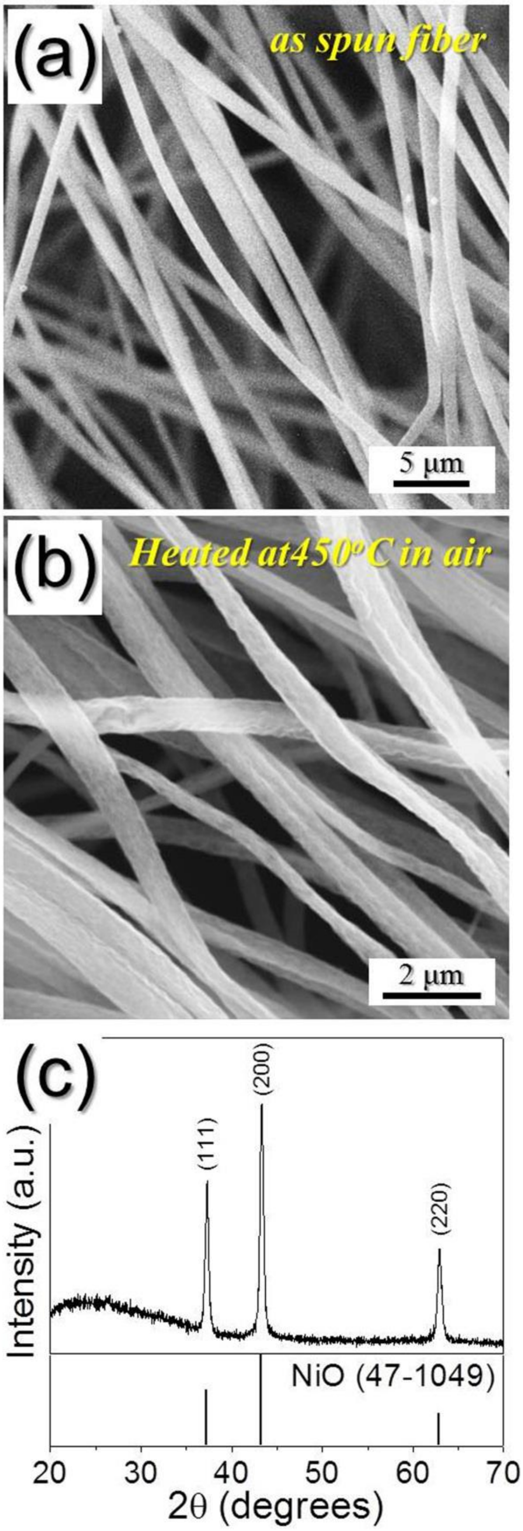

Figure S2 Morphologies and XRD pattern of the intermediate structures of the bare $NiSe_2$ nanofibers. SEM images of the nanofibers (a) before heat treatment, (b) after heat treatment at 450 ^{o}C in air, and (c) XRD pattern of the nanofibers after heat treatment at 450 ^{o}C in air.

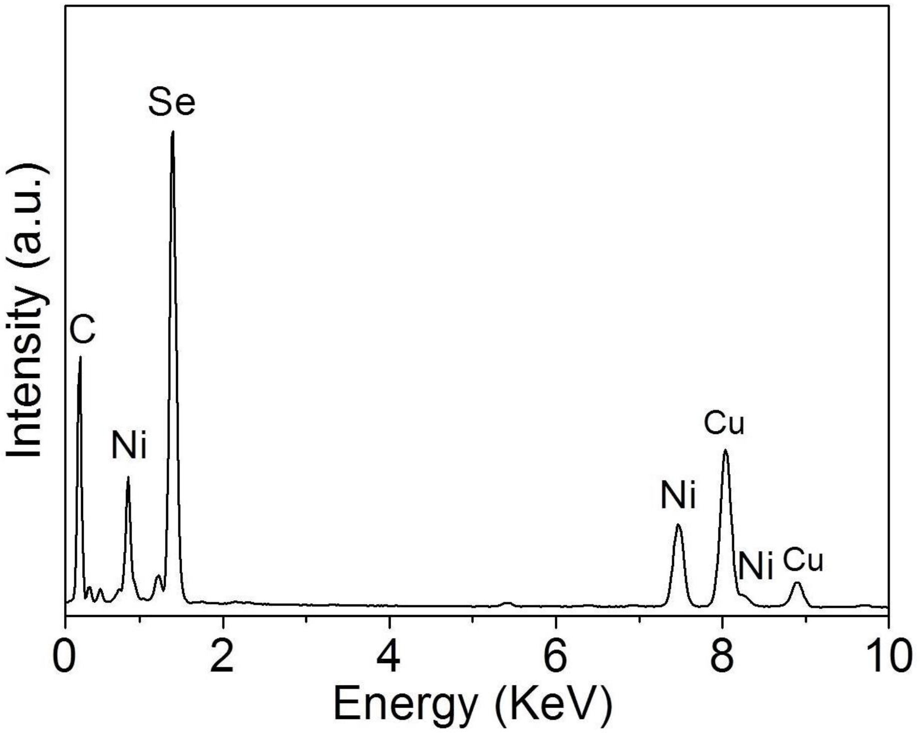

Figure S3 Energy dispersive spectroscopy (EDS) analysis of the $NiSe_2$-rGO-C composite nanofibers formed by selenization process.

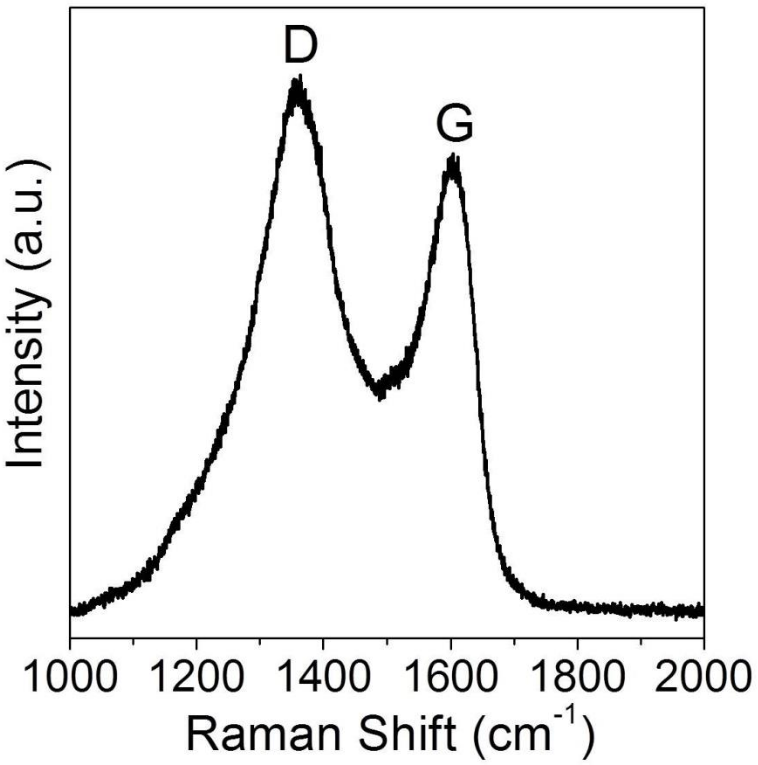

Figure S4 Raman spectrum of the $NiSe_2$-rGO-C composite nanofibers formed by selenization process.

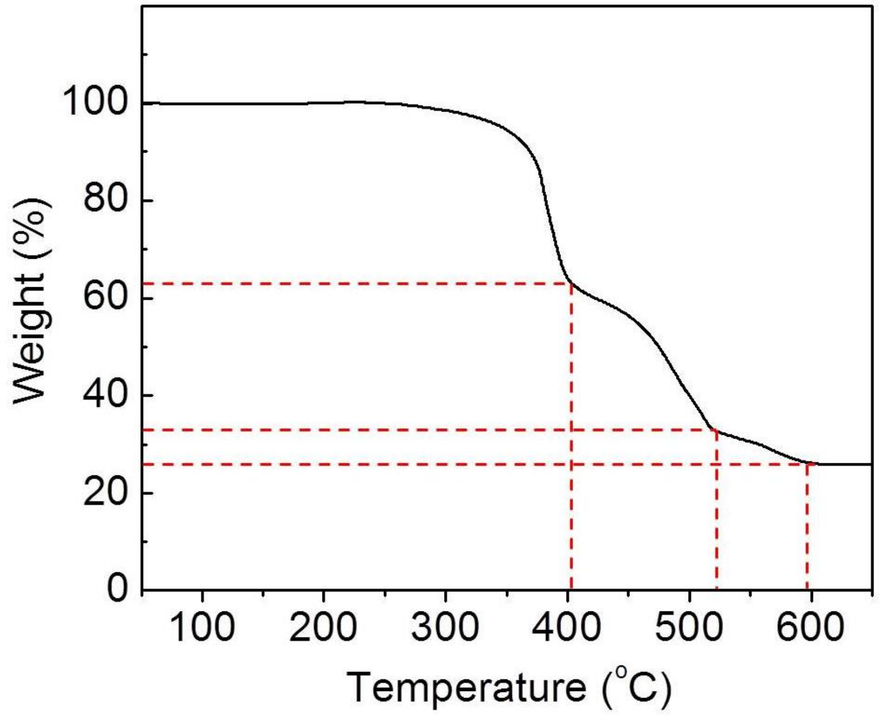

Figure S5 TG analysis of the $NiSe_2$-rGO-C composite nanofibers formed by selenization process.

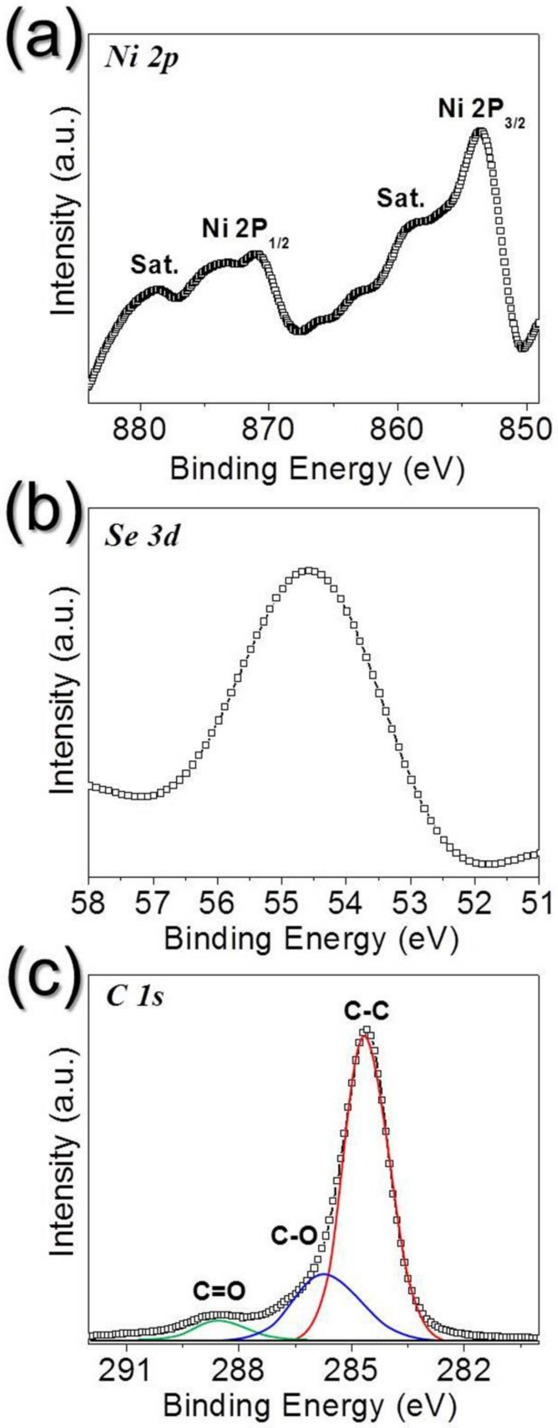

Figure S6 XPS spectra of the $NiSe_2$-rGO-C composite nanofibers formed by selenization process. (a) Ni 2p, (b) Se 3d, and (c) C 1s.

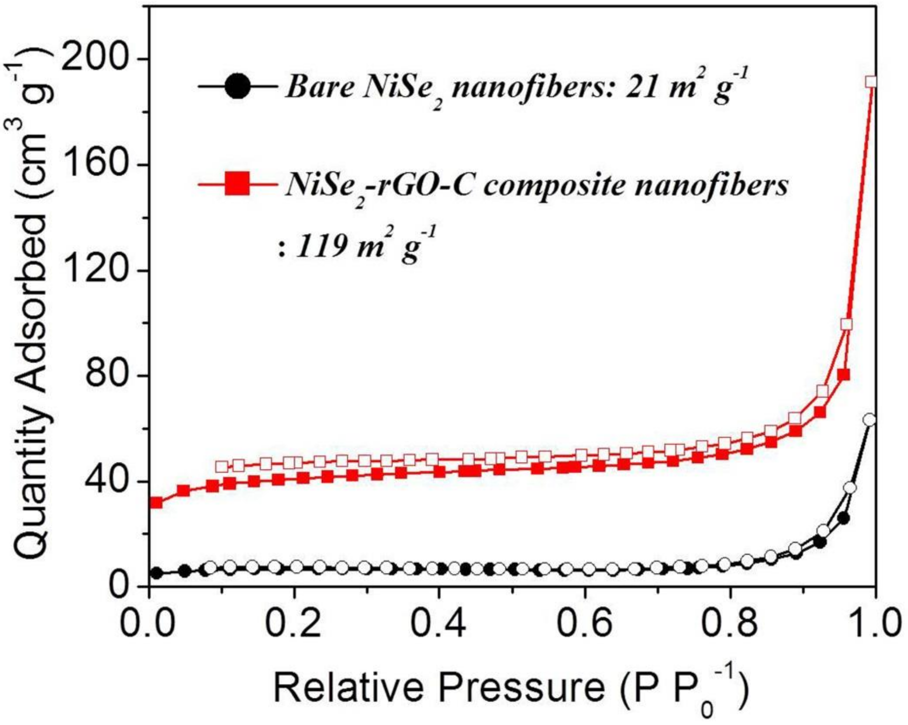

Figure S7 N_2 adsorption-desorption isotherms measured at 77 K for the $NiSe_2$-rGO-C composite and bare $NiSe_2$ nanofibers.

Table S1. Electrochemical properties of the nickel compound materials with various structures as anode materials for NIBs.

Materials	Voltage Range	Current Density	Initial Coulombic Efficiency	Initial Discharge/Charge Capacity	Last Discharge Capacity	Cycle Number	Ref
$NiSe_2$-rGO-carbon composite nanofiber	0.001–3.0 V	200 mA g^{-1}	72 %	717 mA h g^{-1}/ 516 mA h g^{-1}	468 mA h g^{-1}	100	This work
NiO-film	0.005-3.0 V	100 mA g^{-1}	~71 %	744 mA h g^{-1}/ 550 mA h g^{-1}	~215 mA h g^{-1}	100	S1
NiS nano rods-rGO	0.005-3.0 V	50 mA g^{-1}	~75 %	701 mA h g^{-1}/ 524 mA h g^{-1}	~500 mA h g^{-1}	3	S2
Ni_3S_2 powders	0.04-2.6 V	50 mA g^{-1}	90 %	420 mA h g^{-1}/ 376 mA h g^{-1}	342 mA h g^{-1}	15	S3
Ni_3S_2 powders	0.4-2.6 V	450 mA g^{-1}	-	430 mA h g^{-1}	220 mA h g^{-1}	100	S4
Monolithic Ni_3S_2	0.5-2.5 V	20 μA cm^{-2}	-	220 μA h cm^{-2}	~170 μA h cm^{-2}	20	S5
Layered nickel sulfide-rGO	0.005-3.0 V	100 mA g^{-1}	80 %	665 mA h g^{-1}/ 529 mA h g^{-1}	392 mA h g^{-1}	50	S6

References

S1. Jiang, Y. *et al*. Transition metal oxides for high performance sodium ion battery anodes. *Nano Energy* **5**, 60–66 (2014).

S2. Pan, Q. *et al*. Reduced graphene oxide-induced recrystallization of NiS nanorods to nanosheets and the improved Na-storage properties. *Inorg. Chem.* **53**, 3511–3518 (2014).

S3. Kim, J. S. *et al.* The discharge properties of Na/Ni_3S_2 cell at ambient temperature. *J. Power Sources* **178**, 852–856 (2008).

S4. Ryu, H. S. *et al*. Degradation mechanism of room temperature Na/Ni_3S_2 cells using Ni_3S_2 electrodes prepared by mechanical alloying. *J. Power Sources* **244**, 764–770 (2013).

S5. Go, D. Y. *et al*. Electrochemical properties of monolithic nickel sulfide electrodes for use in sodium batteries. *Mater. Res. Bull.* **58**, 190–194 (2014).

S6. Qin, W., Chen, T., Lu, T., Chua, D. H. & Pan, L. Layered nickel sulfide-reduced graphene oxide composites synthesized via microwave-assisted method as high performance anode materials of sodium-ion batteries. *J. Power Sources* **302**, 202–209 (2016).
